# Journal Change

**Published:** 1895-12

**Authors:** 


### JOURNAL CHANGE.

The following letter of one of the editors and owners of the Journal severing his connection with the ownership and business management explains itself. We regret very much that business circumstances cause us this loss, but we are assured that he will continue a contributor to our columns, and thus our readers will not lose the value of his experience and writings, which we are sure will be fully appreciated by all:

Dr. W. Horace Hoskins,
Managing Editor of the Journal of Comparative Medicine, Philadelphia.

Dear Doctor: In severing my business connection with the Journal I feel that I owe you and the readers of the Journal a statement of facts in an open letter. About one year and a half ago, knowing how irregular the publication of the Journal was becoming and feeling that the profession needed some medium for the interchange of views which would be in no way connected with any institution of learning, it occurred to me that the infusion of new blood might be of benefit to the JOURNAL, and consequently to the profession. I offered assistance, which was accepted, and suggested that you be asked to coöperate. The result of several conferences was expressed in the first publication under the new management last fall. I do not think the JOURNAL suffered any by the change. I do not think it would have improved any had we not been fortunate enough to have enlisted your enthusiasm. For my part I have failed sadly in carrying out my part of the contract. I freely admit that I did not anticipate the amount of work and the continual drain of one's purse necessary in the publication of a veterinary journal. Circumstances have sometimes made the necessary expenditure of time and money embarrassing even in my case—how much more so in your case I can hardly imagine. I am unwilling to have my name appear longer as at present, carrying the implication that I am assuming one-fourth or one-fifth of the responsibility, because I am not, neither have I at any time. The credit of having brought up the JOURNAL to its present standard is due to you, and the profession is certainly to be congratulated that within its ranks is to be found one who is willing to sacrifice so much of his time and money for the benefit of the profession. Please do not think for one moment that my withdrawal from the management will have any influence upon my interest in the JOURNAL, for I assure you it will not. Indeed, I think that I can be of more assistance than ever. At least I shall try.

Yours truly,

A. W. CLEMENT.

916 CATHEDRAL STREET, BALTIMORE, MD., November 20, 1895.

